# Left Atrial Intimal Sarcoma Masquerading as Intracardiac Thrombus

**DOI:** 10.7759/cureus.114870

**Published:** 2026-08-20

**Authors:** Hisham B Sweidan, Feng Zhu

**Affiliations:** 1 Internal Medicine, University of Iowa Hospitals and Clinics, Iowa City, USA; 2 Internal Medicine, College of Medicine, University of Arizona, Tucson, USA

**Keywords:** atrial mass, atrial thrombosis, cardiac sarcoma, primary cardiac malignancy, sarcoma

## Abstract

Primary cardiac malignancies are rare and often present with non-specific clinical and imaging findings, leading to diagnostic delays. We report a case of a 76-year-old woman with progressive dyspnea who was found to have a left atrial mass initially suspected to be a thrombus or benign lesion because of the absence of significant uptake on fluorodeoxyglucose positron emission tomography (FDG-PET). However, progressive enlargement and infiltrative imaging features raised concern for malignancy. Histopathological evaluation ultimately confirmed undifferentiated pleomorphic sarcoma with MDM2 amplification, consistent with intimal sarcoma. This case highlights the limitations of FDG-PET in excluding cardiac malignancy and underscores the importance of integrating multimodal imaging with clinical suspicion to avoid diagnostic anchoring.

## Introduction

Primary cardiac tumors are exceedingly uncommon, with an estimated incidence of less than 0.1% in autopsy studies [1]. Among these, approximately 25% are malignant, with sarcomas representing the majority [2]. Intimal sarcoma is a rare and aggressive subtype characterized by rapid growth, local invasion, and early metastasis [3].

The diagnostic evaluation of intracardiac masses is complex. Thrombus, myxoma, and malignant tumors may share overlapping imaging characteristics. While fluorodeoxyglucose positron emission tomography (FDG-PET) is often used to distinguish benign from malignant lesions, its sensitivity is variable, and false-negative results can occur, particularly in tumors with low metabolic activity or necrotic components [4,5].

Clinically, atrial intimal sarcoma often presents with non-specific symptoms such as dyspnea, fatigue, chest discomfort, or signs of heart failure, primarily due to obstruction of intracardiac blood flow or involvement of valvular structures [6]. In cases involving the left atrium, tumor extension toward the mitral valve apparatus can lead to elevated left-sided filling pressures and secondary pulmonary hypertension. Due to the overlap of these symptoms with more common cardiovascular conditions, diagnosis is frequently delayed. In addition, these tumors may resemble benign intracardiac lesions such as myxomas on initial evaluation, further complicating early recognition [7].

## Case presentation

A 76-year-old woman with a history of tachycardia-bradycardia syndrome, status post dual-chamber pacemaker placement, and paroxysmal atrial fibrillation presented with progressive dyspnea on exertion. She reported chronic symptoms for over two years, with recent worsening. She denied chest pain, fevers, chills, or night sweats. Her weight was initially stable.

Transthoracic echocardiography in April 2024 demonstrated preserved left ventricular systolic function (ejection fraction 60%), moderate-to-severe mitral regurgitation, severe pulmonary hypertension (estimated pulmonary artery systolic pressure 89 mmHg), and a 2.4-cm echodense mass in the left atrium.

At this stage, the differential diagnosis for a left atrial mass included thrombus, atrial myxoma, primary cardiac malignancy, and metastatic disease. Given the presence of atrial fibrillation and the location of the mass, thrombus was considered the most likely diagnosis.

Transesophageal echocardiography performed in July 2024 demonstrated a heterogeneous 3-cm mass extending into the left atrial appendage, with internal Doppler flow (Videos 1-3). The morphology was not typical of atrial myxoma. Internal vascularity raised concern for neoplasm; however, imaging was not definitive.

**Video 1:** Initial transesophageal echocardiography demonstrating an approximately 3-cm heterogeneous left atrial mass (arrow) at a multiplane angle of 0° on presentation. TEE: transesophageal echocardiography

**Video 2:** Initial transesophageal echocardiography at a multiplane angle of 33° provides an alternative view of the heterogeneous left atrial mass, highlighting its irregular contour and intracavitary extent. TEE: transesophageal echocardiography

**Video 3:** Initial transesophageal echocardiography demonstrating the multilobulated morphology of the left atrial mass and its apparent broad-based involvement of the interatrial septum at a multiplane angle of -4° on presentation. TEE: transesophageal echocardiography

**Figure 1:**
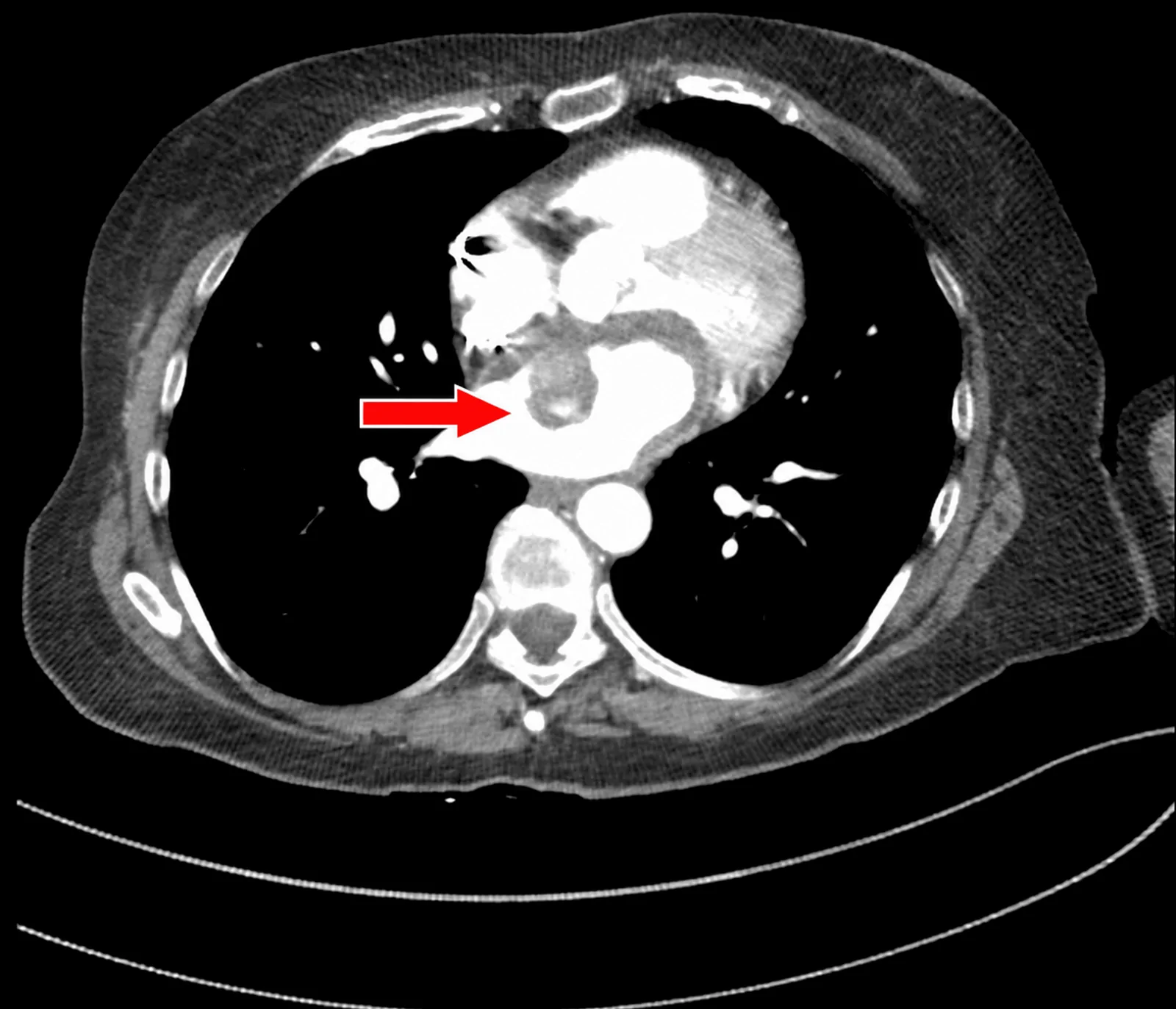
CT angiography of the chest demonstrating the mass. Axial image demonstrates a large, heterogeneous hypoattenuating mass within the contrast-opacified left atrium (arrow), occupying a substantial portion of the atrial cavity.

Further evaluation with computed tomography angiography in October 2024 demonstrated a large infiltrative left atrial mass concerning malignancy, along with multiple sclerotic osseous lesions (Figure 1). These findings raised suspicion for metastatic disease. However, PET/CT revealed that the intracardiac mass was non-FDG-avid (photopenic), whereas the osseous lesions demonstrated increased FDG uptake.

This discordance complicated the diagnostic process. The lack of FDG uptake within the cardiac mass favored thrombus, while the FDG-avid bone lesions suggested a separate malignant process. Bone marrow biopsy and targeted osseous biopsy demonstrated non-specific fibroinflammatory changes without evidence of malignancy. Given the presumed diagnosis of thrombus, a transseptal biopsy was planned but deferred in February 2025 after repeat imaging suggested a decrease in the size of the atrial mass. This apparent regression reinforced the working diagnosis of thrombus.

**Figure 2:**
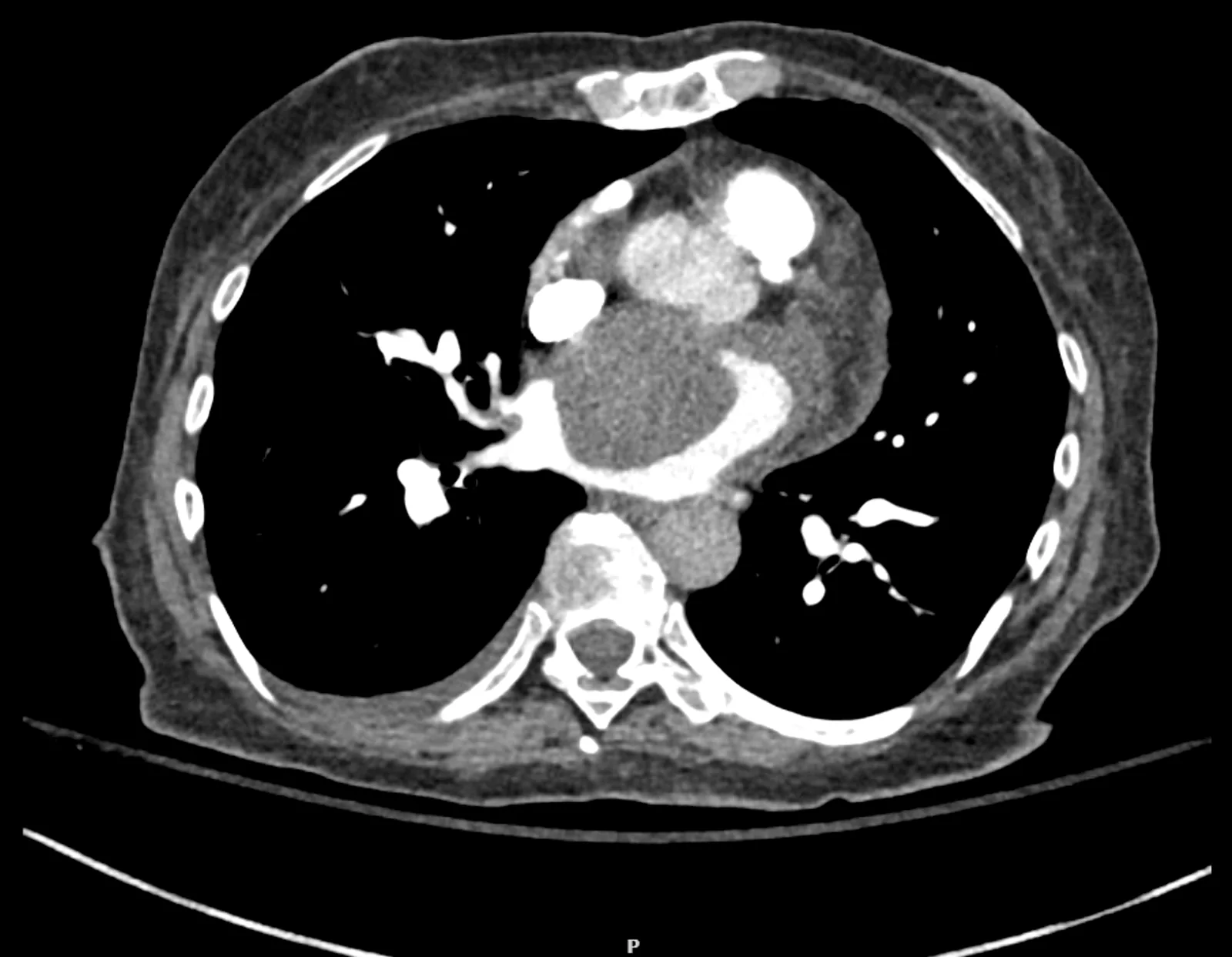
A repeat CT angiography chest. Repeat CT angiography demonstrates progression of the left atrial mass. Axial contrast-enhanced image demonstrates a large, heterogeneous hypoattenuating mass measuring approximately 5.2 cm in maximal dimension, occupying most of the left atrial cavity. The mass is lobulated and has a broad interface with the atrial wall, with marked narrowing of the residual contrast-opacified atrial lumen. These findings, together with the substantial interval enlargement from earlier imaging, favor an infiltrative cardiac neoplasm over organized thrombus.

In April 2025, the patient presented with worsening dyspnea, weight loss, anemia, and systemic inflammation. Repeat computed tomography showed interval enlargement of the left atrial mass to 5.2 cm (Figure 2). The increase in size despite presumed thrombus raised concern for an alternative diagnosis. Another transesophageal echocardiogram was obtained (Videos 4, 5). Her home Eliquis was held, and the patient was placed on a heparin drip.

**Video 4:** Follow-up transesophageal echocardiography obtained approximately six months after the initial examination, demonstrating marked enlargement of the heterogeneous left atrial mass (arrow). TEE: transesophageal echocardiography

**Video 5:** Follow-up transesophageal echocardiography at a multiplane angle of 0° demonstrates marked interval enlargement of the heterogeneous left atrial mass with extensive intracavitary involvement compared with the initial examination (arrow). TEE: transesophageal echocardiography

**Figure 3:**
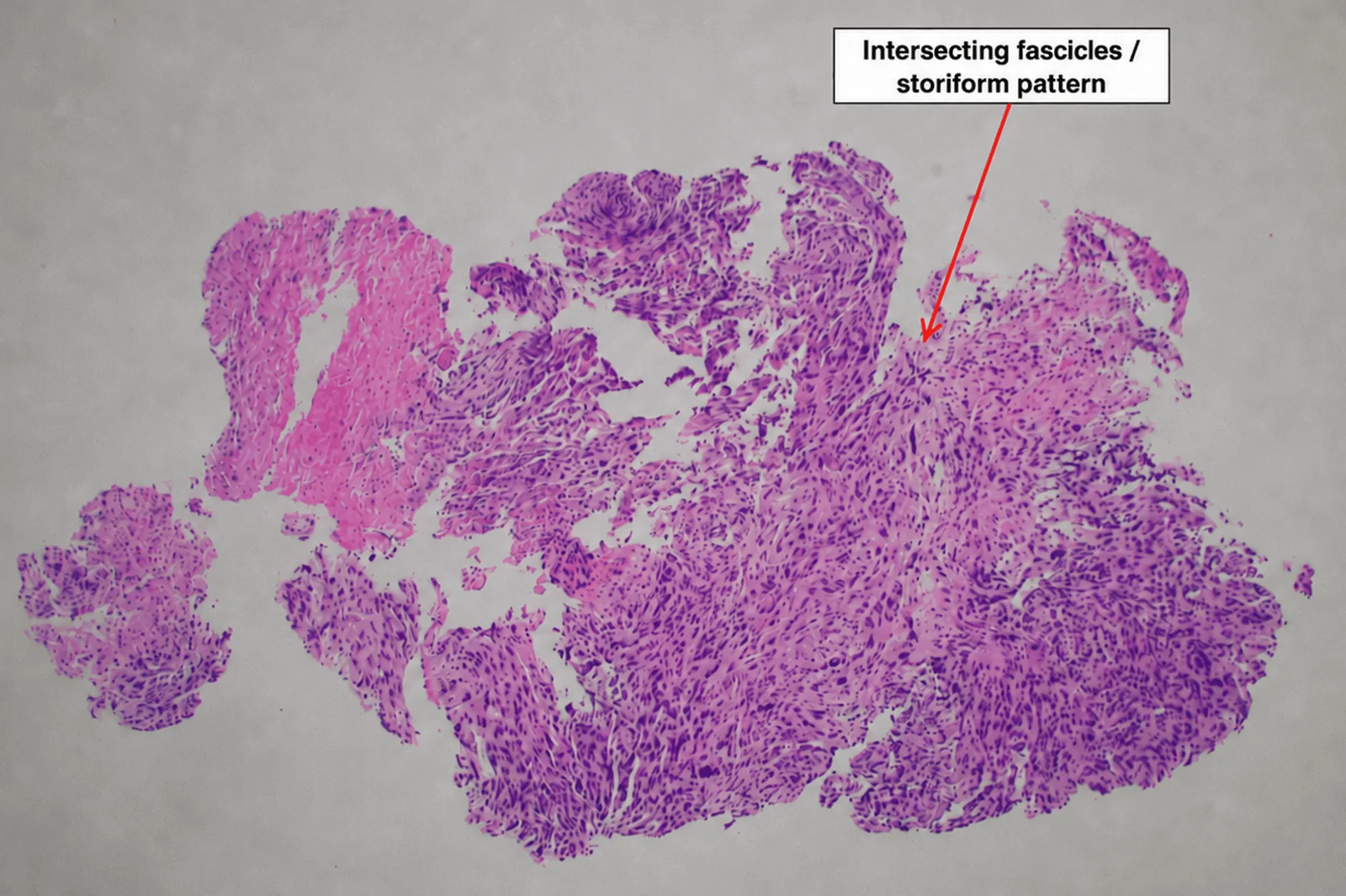
Hematoxylin and eosin (H&E)-stained section. Hematoxylin and eosin (H&E)-stained section demonstrating a high-grade malignant pleomorphic spindle cell neoplasm composed of intersecting fascicles and sheets of markedly atypical spindle to pleomorphic cells with a storiform growth pattern (arrow) (low-power view).

**Figure 4:**
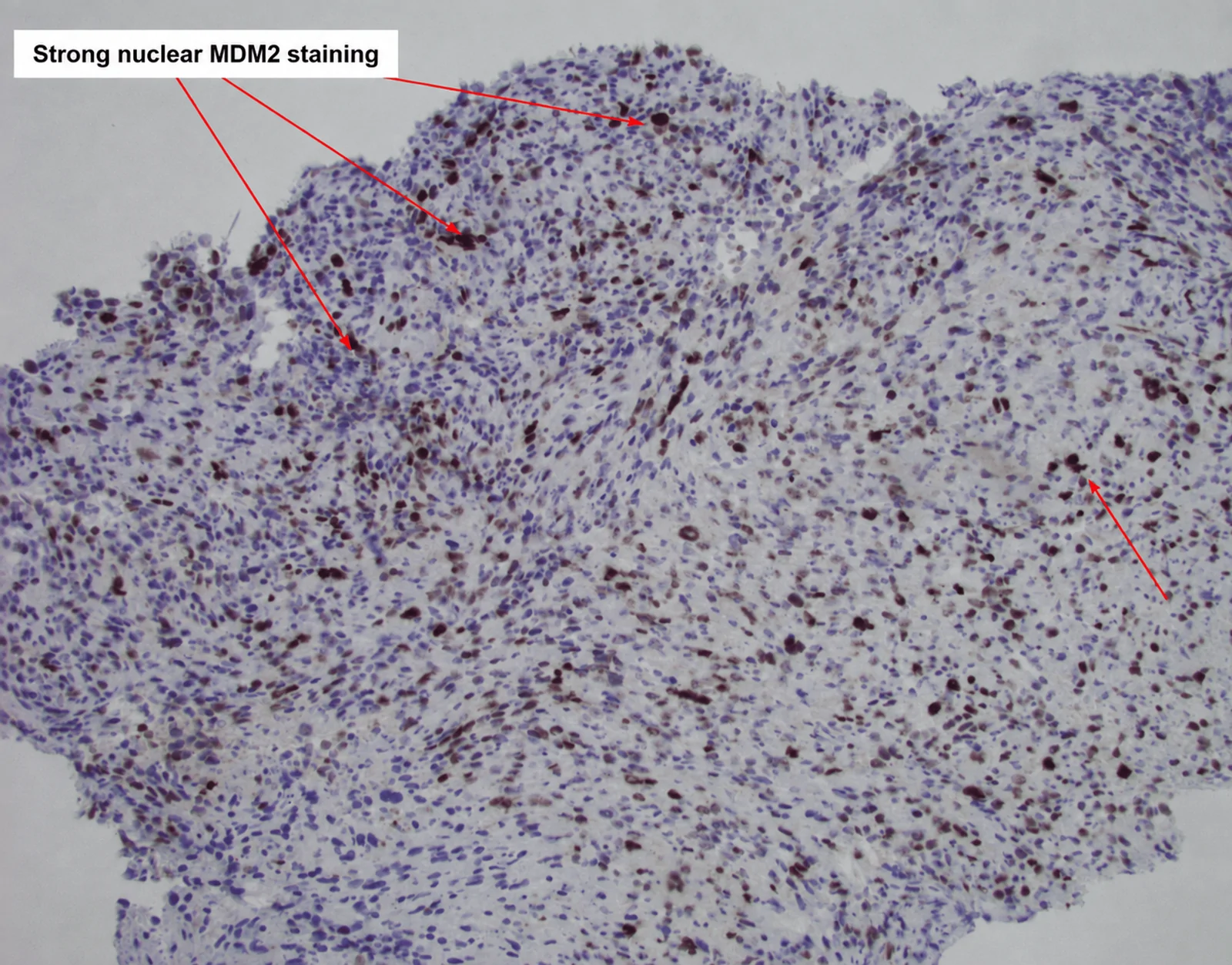
MDM2 immunohistochemical staining demonstrates strong nuclear staining in neoplastic spindle and pleomorphic cells, supporting MDM2 overexpression/amplification and the diagnosis of intimal sarcoma. MDM2 amplification refers to an increased number of copies of the MDM2 gene, which can increase MDM2 protein production and suppress the p53 tumor-suppressor pathway; this molecular alteration is characteristic of intimal sarcoma and supports its diagnosis.

A transseptal biopsy of the left atrial mass was performed. Histopathology demonstrated an undifferentiated pleomorphic sarcoma with MDM2 amplification by immunohistochemistry, consistent with intimal sarcoma (Fédération Nationale des Centres de Lutte Contre le Cancer {FNCLCC} grade 3) (Figures 3, 4).

Over the following weeks, the patient experienced rapid clinical deterioration, including worsening dyspnea, metabolic derangements, and functional decline. Palliative radiation therapy was initiated. However, her condition continued to worsen, and she was transitioned to hospice care, where she subsequently passed away. The chronological sequence of the patient’s presentation, diagnostic evaluation, disease progression, and clinical outcome is summarized in Table 1.

**Table 1:** The chronological events of the patient's presentation until the outcome. TTE: transthoracic echocardiography; EF: ejection fraction; PVC: premature ventricular contraction; FDG: fluorodeoxyglucose; FNCLCC: Fédération Nationale des Centres de Lutte Contre le Cancer

Year	Timeline	Clinical events and findings
2022	May 2022	TTE: EF 50-55%, no intracardiac mass, grade 2 diastolic dysfunction
2023	January 2023	Evaluation for bradycardia and PVCs
	March 2023	Permanent pacemaker placement - new diagnosis: paroxysmal atrial fibrillation → anticoagulation initiated
	2023	Stable symptoms with mild dyspnea
2024	April 2024	Progressive dyspnea (TTE): left atrial mass (2.4×2.1 cm), severe mitral regurgitation, pulmonary hypertension
	July 2024	TEE: Large heterogeneous left atrial mass (3×2 cm), extension into left atrial appendage
	October 2024	CT showed an infiltrative left atrial mass and bone lesions involving T7 and the pelvis. PET showed no FDG uptake in the cardiac mass but demonstrated FDG-avid lymph node and bone lesions
	November 2024	Bone marrow biopsy: non-diagnostic, soft tissue biopsy: non-diagnostic
2025	February 2025	Transseptal biopsy was planned but deferred after repeat imaging suggested a decrease in the mass size, reinforcing the presumed diagnosis of thrombus
	April 2025	Rapid tumor progression (2.9×5.1 cm), worsening dyspnea requiring hospitalization
	May 2025	Transseptal biopsy demonstrated undifferentiated pleomorphic sarcoma with MDM2 amplification by immunohistochemistry, consistent with FNCLCC grade 3 intimal sarcoma
	May 2025	Initiation of palliative radiation therapy
	Late May 2025	Clinical deterioration followed by transition to hospice care and death

## Discussion

Intimal sarcoma is a rare but highly aggressive primary cardiac malignancy, most frequently involving the left atrium and associated with rapid disease progression and early systemic spread [8]. In a retrospective series of 42 patients with primary cardiac sarcoma, multimodality treatment was associated with a median survival of 36.5 months, compared with 14.1 months among patients receiving a single treatment modality (p=0.05) [9]. These tumors arise from mesenchymal cells of the endocardium and are characterized histologically by high-grade pleomorphic features. A defining molecular hallmark is MDM2 amplification, which promotes tumor proliferation by inhibiting the p53 tumor suppressor pathway and serves as an important diagnostic and potentially therapeutic target [10,11].

The clinical manifestations of atrial sarcoma are often non-specific and largely reflect the tumor’s mechanical and hemodynamic effects. Patients commonly present with dyspnea, fatigue, or signs of congestive heart failure due to obstruction of intracardiac blood flow or involvement of valvular structures [12]. In left atrial tumors, extension toward the mitral valve can impair ventricular inflow and contribute to elevated pulmonary pressures, leading to progressive respiratory symptoms.

A key feature of intimal sarcoma is its aggressive biological behavior. These tumors exhibit rapid growth and a marked tendency for early metastasis. The most frequently involved metastatic sites include the lungs, bones, and lymph nodes [13]. The presence of extracardiac disease at diagnosis is common and reflects advanced-stage malignancy, which significantly limits therapeutic options and contributes to poor clinical outcomes.

Imaging is essential in defining tumor extent and guiding management. Echocardiography typically demonstrates a heterogeneous intracardiac mass, while CT and MRI provide additional detail regarding invasion into adjacent structures and overall tumor burden [4]. These tumors often display infiltrative margins and internal heterogeneity, including areas of necrosis or hemorrhage, consistent with high-grade sarcomatous pathology. Functional imaging may provide complementary information, although metabolic activity can vary depending on tumor composition [14].

Management of cardiac intimal sarcoma remains challenging. Surgical resection, when feasible, offers the best opportunity for improved survival and remains the cornerstone of treatment [15]. However, complete resection is often not possible due to extensive local invasion or metastatic disease at the time of diagnosis [16]. In such cases, systemic therapies and radiation are typically employed for palliation, though their impact on long-term survival remains limited.

Despite advances in diagnostic and therapeutic approaches, prognosis remains poor. The reported median survival is generally less than one year, particularly in patients with advanced or unresectable disease [15,17]. The clinical course observed in this case is consistent with the known aggressive natural history of this malignancy.

In summary, atrial intimal sarcoma is a rare and highly malignant tumor characterized by rapid progression, early dissemination, and limited therapeutic options. These primary cardiac tumors are associated with poor outcomes, with reported survival frequently less than one year [18]. Recognition of their clinical, imaging, and molecular features is essential, as timely diagnosis and intervention, when feasible, may offer the only opportunity to improve outcomes.

## Conclusions

Atrial intimal sarcoma is a rare and highly aggressive primary cardiac malignancy characterized by rapid growth, early metastatic dissemination, and poor overall prognosis. Its clinical presentation is often non-specific and driven by intracardiac obstruction and hemodynamic compromise, which can delay recognition. Diagnosis relies on a combination of imaging and histopathological confirmation, with identification of MDM2 amplification supporting the diagnosis.

Despite advances in diagnostic imaging and therapeutic strategies, outcomes remain limited, particularly in patients presenting with advanced or metastatic disease. Surgical resection, when feasible, offers the best opportunity for improved survival, although complete excision is frequently not achievable. This case highlights the aggressive natural history of atrial intimal sarcoma and underscores the importance of early recognition and timely intervention when possible.

## References

[1] Lam KY, Dickens P, Chan AC (1993). Tumors of the heart. A 20-year experience with a review of 12,485 consecutive autopsies. Arch Pathol Lab Med.

[2] Reynen K (1996). Frequency of primary tumors of the heart. Am J Cardiol.

[3] Burke A, Virmani R (1996). Tumors of the heart and the great vessels. Atlas of Tumor Pathology. Third Edition.

[4] Motwani M, Kidambi A, Herzog BA, Uddin A, Greenwood JP, Plein S (2013). MR imaging of cardiac tumors and masses: a review of methods and clinical applications. Radiology.

[5] Rahbar K, Seifarth H, Schäfers M (2012). Differentiation of malignant and benign cardiac tumors using 18F-FDG PET/CT. J Nucl Med.

[6] Hwang JW (2020). A rare disease of primary cardiac intimal sarcoma at the left atrium presented with mimicking mitral stenosis in a patient with polycystic kidney disease. J Cardiol Cases.

[7] Isambert N, Ray-Coquard I, Italiano A (2014). Primary cardiac sarcomas: a retrospective study of the French Sarcoma Group. Eur J Cancer.

[8] Neuville A, Collin F, Bruneval P (2014). Intimal sarcoma is the most frequent primary cardiac sarcoma: clinicopathologic and molecular retrospective analysis of 100 primary cardiac sarcomas. Am J Surg Pathol.

[9] Randhawa JS, Budd GT, Randhawa M (2016). Primary cardiac sarcoma: 25-year Cleveland Clinic experience. Am J Clin Oncol.

[10] Nistor C, Carsote M, Cucu AP, Stanciu M, Popa FL, Ciuche A, Ciobica ML (2024). Primary cardiac intimal sarcoma: multi-layered strategy and core role of MDM2 amplification/co-amplification and MDM2 immunostaining. Diagnostics (Basel).

[11] Grant L, Morgan I, Sumathi V, Salmons N (2019). Intimal sarcoma of the left atrium presenting with transient ischaemic attack - a case report and review of the literature. J Cardiol Cases.

[12] Yusuf SW, Bathina JD, Qureshi S (2012). Cardiac tumors in a tertiary care cancer hospital: clinical features, echocardiographic findings, treatment and outcomes. Heart Int.

[13] Blackmon SH, Reardon MJ (2009). Surgical treatment of primary cardiac sarcomas. Tex Heart Inst J.

[14] Nensa F, Tezgah E, Poeppel TD (2015). Integrated 18F-FDG PET/MR imaging in the assessment of cardiac masses: a pilot study. J Nucl Med.

[15] Ramlawi B, Leja MJ, Saleh WK (2016). Surgical treatment of primary cardiac sarcomas: review of a single-institution experience. Ann Thorac Surg.

[16] Chan EY, Ali A, Zubair MM (2023). Primary cardiac sarcomas: treatment strategies. J Thorac Cardiovasc Surg.

[17] Ho K, Yatham K, Seno R, Sultan O (2021). A case report of primary cardiac intimal sarcoma presenting with atrial fibrillation and a left atrial mass. Eur Heart J Case Rep.

[18] Valecha G, Pau D, Nalluri N, Liu Y, Mohammad F, Atallah JP (2016). Primary intimal sarcoma of the left atrium: an incidental finding on routine echocardiography. Rare Tumors.

